# Early administration of trimetazidine attenuates diabetic cardiomyopathy in rats by alleviating fibrosis, reducing apoptosis and enhancing autophagy

**DOI:** 10.1186/s12967-016-0849-1

**Published:** 2016-04-27

**Authors:** Lei Zhang, Wen-yuan Ding, Zhi-hao Wang, Meng-xiong Tang, Feng Wang, Ya Li, Ming Zhong, Yun Zhang, Wei Zhang

**Affiliations:** The Key Laboratory of Cardiovascular Remodeling and Function Research, Chinese Ministry of Education and Chinese Ministry of Health, the State and Shandong Province Joint Key Laboratory of Translational Cardiovascular Medicine, Jinan, People’s Republic of China; Department of Cardiology, Qilu Hospital of Shandong University, No.107 Wenhua West Road, Jinan, 250012 People’s Republic of China; Department of Cardiology, Shandong Provincial Qianfoshan Hospital, Shandong University, Jinan, 250012 People’s Republic of China; Department of Geriatric Medicine, Qilu Hospital of Shandong University, Jinan, 250012 People’s Republic of China; Department of Emergency, Qilu Hospital of Shandong University, Jinan, 250012 People’s Republic of China

**Keywords:** Diabetic cardiomyopathy, Trimetazidine, Autophagy, Myocardial fibrosis, Apoptosis

## Abstract

**Background:**

Trimetazidine, as an anti-ischemic and antioxidant agent, has been demonstrated to have many cardioprotective effects. However, whether early administration of trimetazidine has an effect on diabetic cardiomyopathy and the mechanisms underlying the effect have not yet been elucidated.

**Methods:**

We established a type 2 DCM rat model by high-fat diet and low-dose streptozotocin. Rats were separated into different groups: control, diabetes, and diabetes + trimetazidine (n = 6, each). Cardiac autophagy, cardiac functions, and cardiomyocyte apoptosis were monitored.

**Results:**

Rats with type 2 DCM showed severe insulin resistance, left ventricular dysfunction, increased cardiomyocyte apoptosis, and reduced cardiac autophagy. Collagen volume fraction (CVF) and perivascular collagen area/luminal area (PVCA/LA) ratio were significantly higher in the diabetic group than the control group. We found that trimetazidine treatment ameliorated metabolic disturbance and insulin resistance, reduced cardiomyocyte apoptosis, and restored cardiac autophagy. CVF and PVCA/LA ratio were also lower in the diabetes + trimetazidine group than the diabetic group (CVF, 4.75 ± 0.52 % vs. 11.04 ± 1.67 %, *p* < 0.05; PVCA/LA, 8.37 ± 0.51 vs. 17.97 ± 2.66, *p* < 0.05). Furthermore, trimetazidine inhibited phosphorylation of ERK and P38 MAPK to reduce myocardial fibrosis. Inhibited phosphorylation of AMPK was restored and the interaction between Bcl-2 and Beclin1 was enhanced in diabetes + trimetazidine group, resulting in the initiation of autophagy and alleviation of apoptosis.

**Conclusions:**

Early administration of trimetazidine could ameliorate diabetic cardiomyopathy by inhibiting myocardial fibrosis and cardiomyocyte apoptosis and enhancing autophagy. Therefore, trimetazidine may be a good choice in the prevention of diabetic cardiomyopathy if applied at the early stage of diabetes.

## Background

Diabetic cardiomyopathy (DCM), which is a major cardiac complication in diabetic patients, is one of the leading causes of increased morbidity and mortality in the diabetic population [1]. It is widely acknowledged that DCM is characterized by a set of structural and functional abnormalities in the heart, including left ventricular dysfunction, cardiomyocyte apoptosis, and myocardial fibrosis [2]. Recent studies have demonstrated that autophagy is also an important contributor to the development and the progression of DCM [3]. Despite lots of researches on the mechanisms of DCM have been reported, there is currently no specific clinical interventions for DCM.

Trimetazidine, which is a first-line anti-anginal agent, selectively inhibits long-chain 3-ketoacyl coenzyme A thiolase (the last enzyme involved in β-oxidation) activity. The mechanism of action of trimetazidine can be attributed to its effect on energy metabolism. In addition to metabolic effects, trimetazidine has been shown to induce autophagy in skeletal muscle myotubes [4], inhibit pressure overload-induced cardiac fibrosis [5], and reduce smoking-induced apoptosis [6]. Although trimetazidine improves ejection fraction (EF) in patients of heart failure with or without ischaemic cardiomyopathy [7–11], the effect of early administration of trimetazidine on DCM and the mechanisms underlying the effect have not yet been elucidated.

Thus, we hypothesized that early administration of trimetazidine has an effect on DCM by inhibiting myocardial fibrosis, reducing cardiomyocyte apoptosis and enhancing autophagic capacity. Thus, we established the type 2 DCM model and gave trimetazidine in vivo to explore the effect of trimetazidine on DCM and the mechanism of this action.

## Methods

### Experiment design

The rat model of type 2 diabetes was established as described previously [12]. Eighteen male Sprague–Dawley rats (120–140 g) were purchased from the experimental animal center of Shandong University of Traditional Chinese Medicine (Ji’nan, China). All rats were randomly assigned to a control group (*n* = 6) or diabetic group (*n* = 12). The diabetic groups were fed a high fat diet (Beijing HFK Bio-Technology, China) and the control group received normal chow. After 4 weeks, the rats with insulin resistance in the diabetic group were given a single intraperitoneal injection of streptozotocin (Sigma, St. Louis, MO; 27.5 mg/kg i.p. in 0.1 mol/L citrate buffer, pH 4.5). One week after streptozotocin administration, rats with plasma glucose levels >11.1 mmol/L were considered a diabetic rat model. The diabetic group (*n* = 12) was redivided into two groups: a diabetes group (*n* = 6) and a diabetes + trimetazidine group (*n* = 6). The diabetes + trimetazidine group was given a dose (30 mg kg^–1^ day^–1^) of trimetazidine (Servier, France) by gavage for 8 weeks. Then rats were sacrificed. The heart was excised and weighed. All animals received humane care and all experimental procedures were performed in accordance with animal protocols approved by the Shandong University Animal Care Committee.

### Glucose and insulin tolerance

An intraperitoneal glucose tolerance test (IPGTT) was carried out to evaluate glucose after rats fasted for 12 h and then injected glucose (1 g/kg) intraperitoneally. Blood glucose level was evaluated at 0, 15, 30, 60, and 120 min postinjection. An intraperitoneal insulin tolerance test (IPITT) was performed to assess insulin tolerance after rats fasted for 4 h and then administered insulin (1 unit/kg) intraperitoneally. Blood glucose level was measured as described above. Blood samples were collected from the tail vein and plasma glucose was measured with a one-touch glucometer (Life Scan, Milpitas, CA). The mean area under the receiver operating characteristic curve (AUC) was calculated for glucose.

### Blood analyses

After rats fasted overnight, blood samples were obtained from the jugular vein. Total cholesterol, triglycerides, fasting blood glucose, HDL-c, and LDL-c were analyzed with use of the Bayer 1650 blood chemistry analyzer (Bayer, Tarrytown, NY). Fasting insulin was measured by enzyme-linked immunosorbent assay. Insulin sensitivity index (ISI) was calculated using the following formula: ISI = ln [([fasting blood glucose] × [fasting insulin])^−1^].

### Measurement of blood pressure

Heart rate, systolic blood pressure, diastolic blood pressure, and mean arterial pressure were measured with a noninvasive tail-cuff system (Softron BP-98A; Softron, Tokyo, Japan) as described previously [13].

### Hemodynamic examination

At the end of the experiments, rats under deep anesthesia underwent hemodynamic measurement. A catheter was inserted from the right common carotid artery into the left ventricle. Left ventricular (LV) end diastolic pressure was measured.

### Histology and morphometric analysis

LV tissues were fixed in 4 % neutral formaldehyde and embedded in paraffin, and 4 μm-thick sections were produced. The sections were stained with HE, Masson-Trichrome, and Picrosirius red. Collagen volume fraction (CVF) and perivascular area/luminal area (PVCA/LA) ratio were obtained by quantitative morphometry with automated image analysis (Image-Pro Plus, Version 5.0; Media Cybernatics, Houston, TX).

### Immunohistochemical staining

Paraffin-embedded tissues were deparaffinized and sections were pretreated by microwaving them for 15 min and were incubated with primary polyclonal anti–collagen I, and anti–collagen III (Abcam, Cambridge, MA) overnight at 4 °C and incubated with horseradish peroxidase-labeled secondary antibody for 1 h at 37 °C. Negative controls were omission of the primary antibody. Finally, the results were viewed under a confocal FV 1000 SPD laser scanning microscope (Olympus, Japan).

### TUNEL assay

TUNEL (terminal deoxynucleotidyl transferase dUTP nick end labeling) detection of apoptotic cells in the hearts was carried out using the ApopTag Peroxidase In Situ Apoptosis Detection Kit (S7101, Chemicon, Billerica, MA, USA). Images of the processed tissues were captured using a confocal FV 1000 SPD laser scanning microscope (Olympus, Japan).

### Western blot analysis

Proteins were extracted from hearts, separated by SDS-PAGE and transferred to polyvinylidene difluoride membrane. The membranes were incubated with a 1:1000 dilution of antibodies for p-AMPK, t-AMPK, Beclin 1, Caspase 3, PARP, Bcl-2, p-ERK, t-ERK, p-P38, t-P38, p-JNK, t-JNK, p-PI3 K, t-PI3K, p-AKT, t-AKT, mTOR, t-mTOR (Cell Signaling Technology, Danves MA, USA), a 1:1000 dilution antibodies for ATG5 and ATG7 (Abcam, Cambridge, MA), and an antibody for Bcl-2 (Biolegend, San Diego, CA) and then 1:10,000 horseradish peroxidase-conjugated secondary antibody. Immunoreactive bands were visualized by use of an enhanced chemiluminescence reagent. Densitometry involved use of photoshop.

### Coimmunoprecipitation assay

Proteins were extracted from hearts and then whole lysate was precleared with protein A/G sepharose beads (Santa Cruz) at 4 °C for 60 min. The cleared supernatant was incubated overnight at 4 °C with the indicated antibodies, followed by incubation for 2 h at 4 °C with protein A/G sepharose beads. Finally, the beads were washed five times, eluted by boiling in sample buffer for SDS-PAGE, and further immunoblot analysis was conducted with the indicated antibodies.

### Statistical analysis

Values are presented as mean ± SEM. SPSS 16.0 (SPSS, Chicago, IL) was used for statistical analysis. Differences between experimental groups were determined by one-way ANOVA or Student’s t test. *p* < 0.05 was considered statistically significant.

## Results

### Generation of type 2 DCM rat model

#### General characteristics of diabetic rats

At the end of the experiment, our results found that water intake, food intake, urine volume, and heart weight were significantly higher in the diabetic group than the control group. There was no difference in blood pressure between the three groups (Table 1).

**Table 1:** Animal characteristics at the end of experiment, according to study group (*n* = 5–6 per group)

Characteristic	Control	Diabetes mellitus	Diabetes mellitus + trimetazidine
Body weight (g)	434.17 ± 8.60	470.17 ± 27.874	433.67 ± 7.893
Heart weight (mg)	1096.00 ± 35.86	1463.33 ± 37.83*	1293.86 ± 47.94**
Water intake (mL/day)	28.20 ± 3.455	153.83 ± 10.329*	147.00 ± 10.440
Food intake (g/day)	14.44 ± 0.423	29.95 ± 1.090*	29.00 ± 1.163
Urine volume (mL/day)	22.60 ± 2.502	126.33 ± 7.766*	129.00 ± 10.416
Systolic blood pressure (mm Hg)	123.40 ± 4.490	120.67 ± 3.964	116.40 ± 5.609
Diastolic blood pressure (mm Hg)	89.00 ± 4.025	77.17 ± 4.888	84.20 ± 2.354
Mean pressure (mm Hg)	103.80 ± 5.229	90.33 ± 5.245	94.80 ± 3.089
Heart rate (bpm)	454.20 ± 15.714	491.67 ± 13.601	343.20 ± 14.235**

Values are mean ± SEM. bpm, beats per minute

*p < 0.05 compared with control group; and **p < 0.05 compared with diabetes group

#### Total cholesterol, triglycerides, fasting blood glucose, HDL-c and LDL-c

**Fig. 1:**
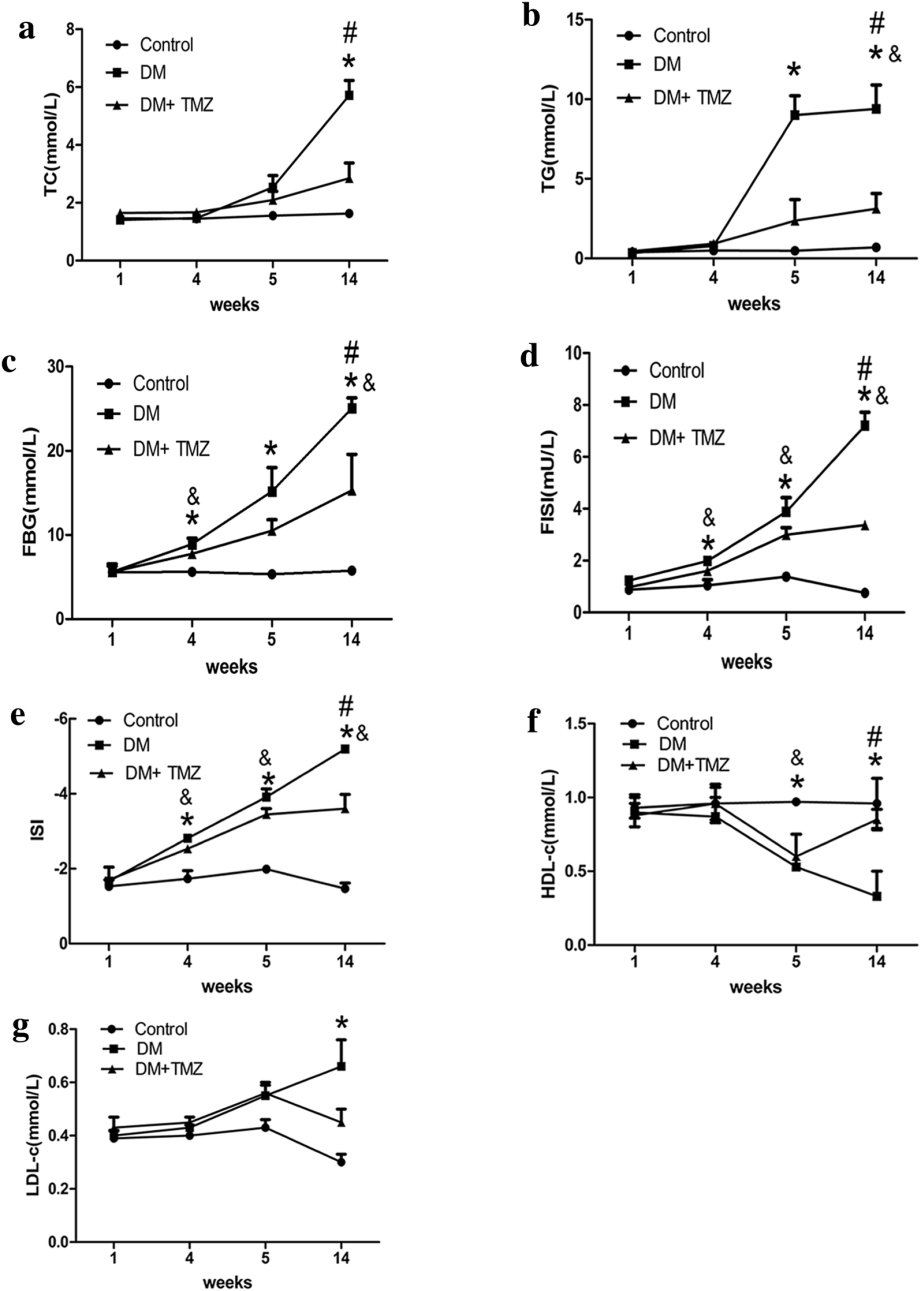
Changes in metabolic indices in rats in the control, diabetes mellitus (DM), and diabetes mellitus + trimetazidine (DM + TMZ) groups (*n* = 5–6 per group). Changes in levels of total cholesterol (TC) (**a**), triglyceride (TG) (**b**), fasting blood glucose (FBG) (**c**), fasting insulin (FINS) (**d**), insulin sensitive index (ISI) (**e**), HDL-c (**f**) and LDL-c (**g**) are shown. Values are mean ± SEM. *p < 0.05 compared with control group; ^#^ *p* < 0.05 compared with diabetes group and ^&^ *p* < 0.05 compared with control group

After 4 weeks of a high fat diet, there was little difference in total cholesterol, triglyceride, HDL-c and LDL-c levels between the diabetic and control groups (Fig. 1a, b, f, g). Serum fasting blood glucose and fasting insulin were significantly higher in the diabetic group than in the control group (fasting blood glucose, 8.93 ± 0.69 vs. 5.61 ± 0.26 mmol/L, *p* < 0.05; and fasting insulin, 1.99 ± 0.97 vs. 1.05 ± 0.22 mU/L, *p* < 0.05, respectively) (Fig. 1c, d). ISI decreased significantly in the diabetic group (−2.81 ± 0.07 vs. −1.72 ± 0.21, *p* < 0.05) (Fig. 1e).

One week after streptozotocin injection (week 5), fasting blood glucose, triglycerides, and fasting insulin were significantly higher in the diabetic group than in the control group (fasting blood glucose, 15.20 ± 2.82 vs. 5.35 ± 0.23 mmol/L, *p* < 0.05; triglycerides, 7.55 ± 1.54 vs. 0.48 ± 0.18 mmol/L; and fasting insulin, 3.88 ± 0.56 vs. 1.37 ± 0.13 mU/L, *p* < 0.05, respectively), and ISI and HDL-c were lower in the diabetic group (ISI, −3.91 ± 0.22 vs. −1.98 ± 0.08, *p* < 0.05; and HDL-c, 0.53 ± 0.11 vs. 0.97 ± 0.01 mmol/L, respectively) (Fig. 1b–f). At the end of the experiment, serum total cholesterol, triglycerides, fasting blood glucose, fasting insulin and LDL-c were significantly higher in the diabetic group than in the control group (total cholesterol, 5.72 ± 0.51 vs. 1.63 ± 0.13 mmol/L; triglycerides, 7.55 ± 0.92 vs. 0.71 ± 0.11 mmol/L; fasting blood glucose, 25.07 ± 1.21 vs. 5.75 ± 0.30 mmol/L; fasting insulin, 7.20 ± 0.51 vs. 0.76 ± 0.13 mU/L, *p* < 0.05; and LDL-c, 0.66 ± 0.1 vs. 0.3 ± 0.03 mmol/L respectively). ISI and HDL-c decreased significantly in the diabetic group (ISI, −5.19 ± 0.19 vs. −1.46 ± 0.15, *p* < 0.05; and HDL-c, 0.33 ± 0.03 vs. 0.96 ± 0.17 mmol/L, respectively). These findings showed the presence of metabolic disturbance and insulin resistance in the diabetic group.

#### Glucose and insulin tolerance

**Fig. 2:**
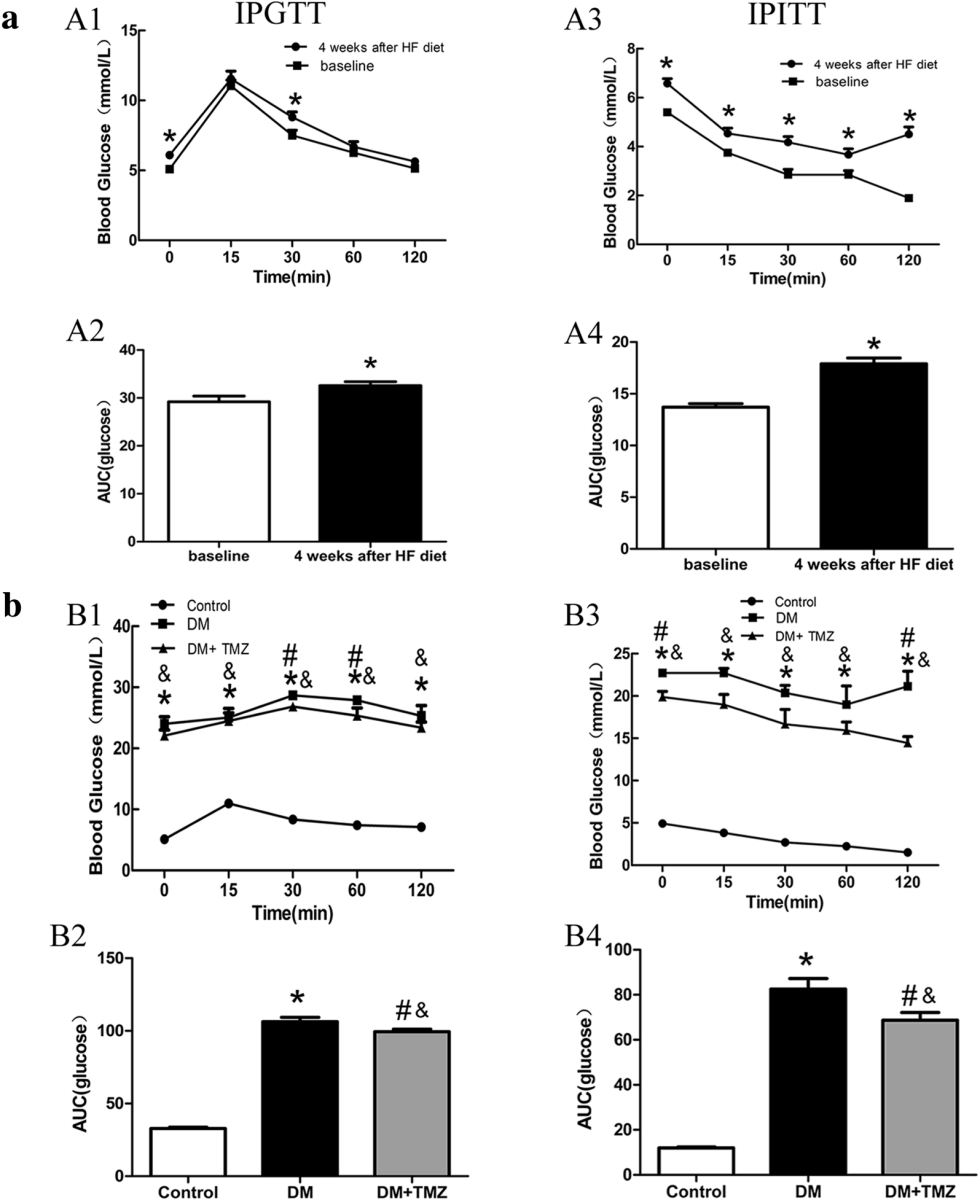
Blood glucose in rats that underwent an intraperitoneal glucose tolerance test (IPGTT) or intraperitoneal insulin tolerance test (IPITT). **a** show blood glucose levels at baseline and after 4 weeks of high fat (HF) diet; while **b** compare blood glucose in rats in the control, diabetes mellitus (DM), and diabetes mellitus + trimetazidine (DM + TMZ) groups (*n* = 5–6 per group) at the end of the experiment. Values are mean ± SEM; *n* = 5–6 per group. *p < 0.05 compared with baseline (**a**) or control group (**b**); ^#^ *p* < 0.05 compared with diabetes group and ^&^ *p* < 0.05 compared with control group

After a 4-week high fat diet, IPGTT and IPITT confirmed the presence of insulin resistance. At week 4, IPGTT revealed that blood glucose levels in the diabetic group were significantly higher at 0 and 30 min than at baseline (Fig. 2A1). The AUC across the time for glucose level was significantly higher at week 4 than at baseline (29.15 ± 0.84 vs. 32.93 ± 2.02,respectively; *p* < 0.05, Fig. 2A2). Similarly, IPITT revealed impaired insulin sensitivity (Fig. 2A3, A4). These data showed that insulin resistance appeared in the diabetic group after 4-week high fat diet. At the end of the experiment, the levels of blood glucose and AUC on both IPGTT and IPITT in the diabetic group were significantly higher than in the control group (Fig. 2B1–B4).

#### Pathology characteristics

**Fig. 3:**
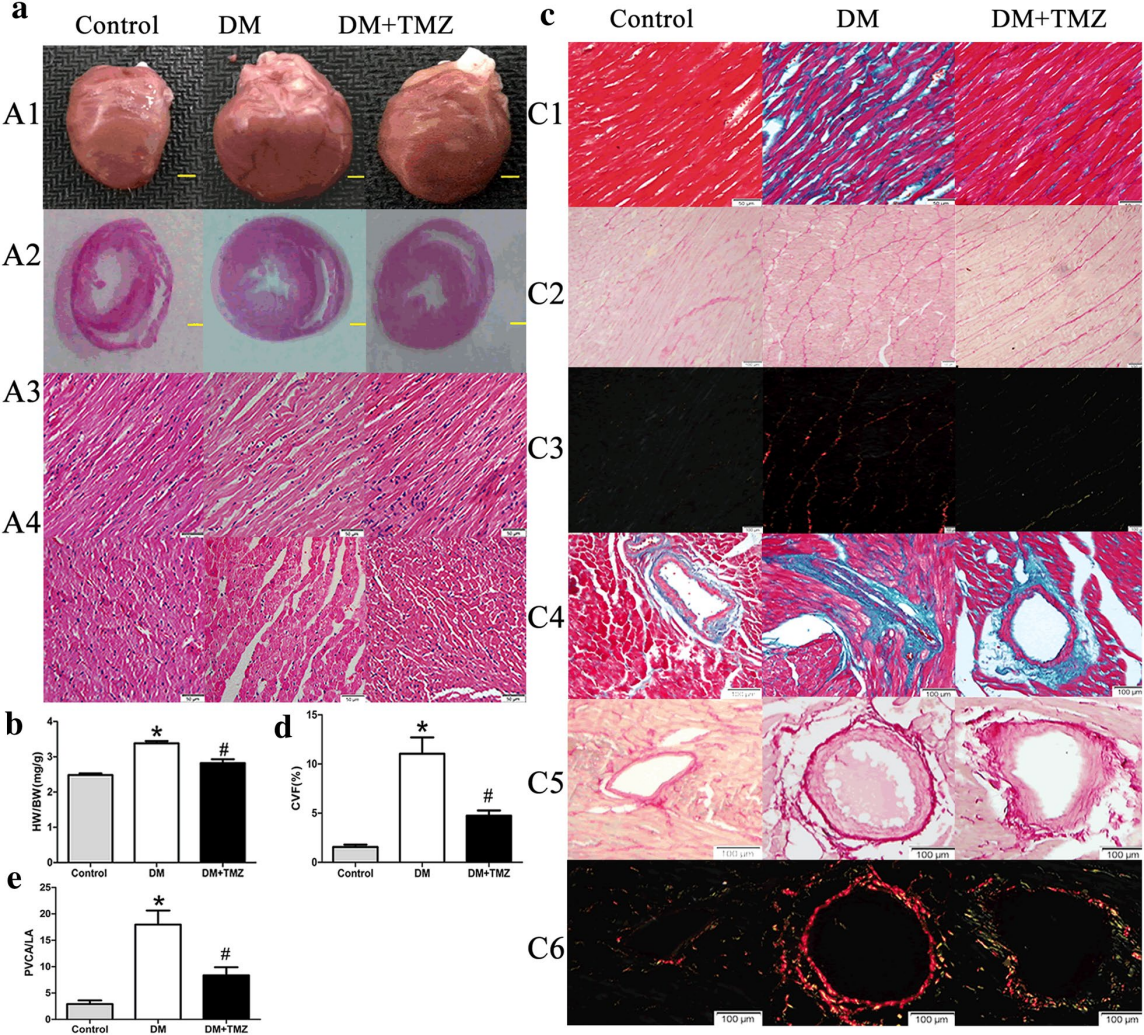
Pathological features of diabetic cardiomyopathy. **a** Cardiac hypertrophy in diabetic rats. Comparison of heart size (*A1*) (*scale bar* 2 mm), representative cross sections of hearts at the papillary muscle level (*A2*) (*scale bar* 2 mm), and longitudinal sections (A3) and transverse sections (*A4*) of left ventricle stained with hematoxylin and eosin (H&E; *scale bar* 50 μm) in rats in the control, diabetes mellitus (DM), and diabetes mellitus + trimetazidine (DM + TMZ) groups (*n* = 5–6 per group). **b** Ratio of heart weight to body weight in rats in the control, DM, and DM + TMZ groups. **c** Cardiac fibrosis in diabetic rats. Interstitial fibrosis as shown by Masson-Trichrome staining [collagen is green and myocardium red; *scale bar* 50 μm (*C1*)] and Picrosirius red staining (collagen fibers stained bright red; bright-field (*C2*) and dark-field (*C3*); *scale bar* 100 μm). Perivascular fibrosis as shown by Masson-Trichrome staining (*C4*) (*scale bar* 100 μm) and Picrosirius red staining [bright-field (*C5*) and dark-field (*C6*); *scale bar* 100 μm]. **d** Comparison of collagen volume fraction (CVF) in rats in the control, DM, and DM + TMZ groups. **e** Comparison of perivascular collagen area/luminal area (PVCA/LA) ratio in rats in the control, DM, and DM + TMZ groups. Values are mean ± SEM. *p < 0.05 compared with control group; and ^ #^ *p* < 0.05 compared with diabetes group

At the end of the experiment, the ratio of heart weight to body weight was higher in the diabetic group than in the control group (3.39 ± 0.07 vs. 2.51 ± 0.05, *p* < 0.05, Fig. 3b). Meanwhile, the diabetic group revealed cardiac fibrosis, with destroyed and irregular collagen network structure in the interstitial and perivascular area. The diabetic group also showed significantly higher CVF (11.04 ± 1.67 % vs. 1.56 ± 0.24 %, *p* < 0.05, Fig. 3d) and PVCA/LA ratio (17.97 ± 2.66 vs. 2.91 ± 0.69, *p* < 0.05, Fig. 3e) compared with the control group.

#### Collagen I and III

**Fig. 4:**
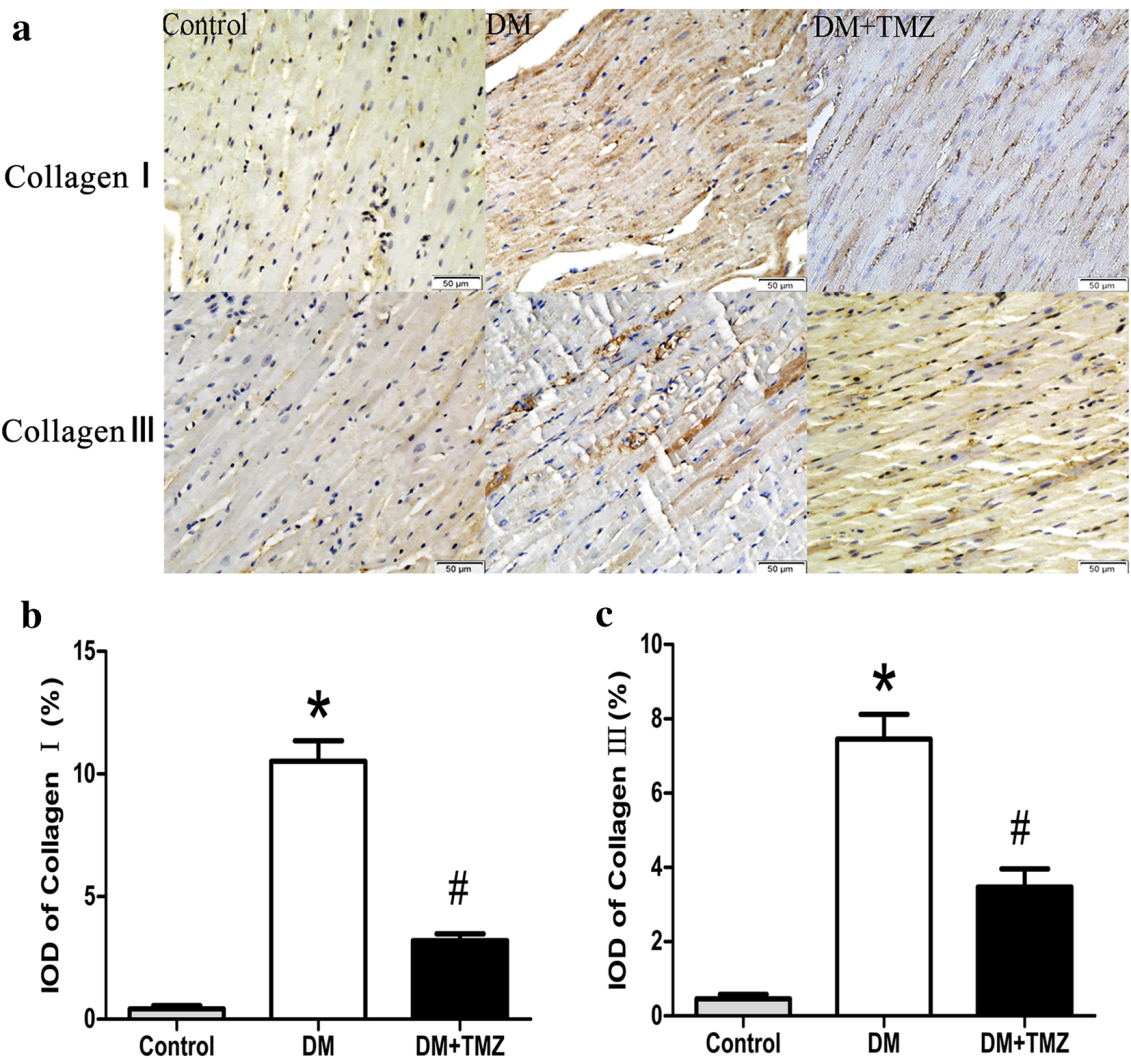
Collagen I and collagen III increased in rats with type 2 diabetes (DM), but decreased in rats with type 2 diabetes given trimetazidine (DM + TMZ). **a**, **b** and **c** show representative immunohistochemical staining of collagen I and III (*scale bar* 50 μm); semiquantification of collagen I staining; and semiquantification of collagen III staining, respectively

Immunohistochemistry showed that the deposition of collagen I and III content was significantly higher in the diabetic group than in the control group (Fig. 4).

#### Apoptosis

**Fig. 5:**
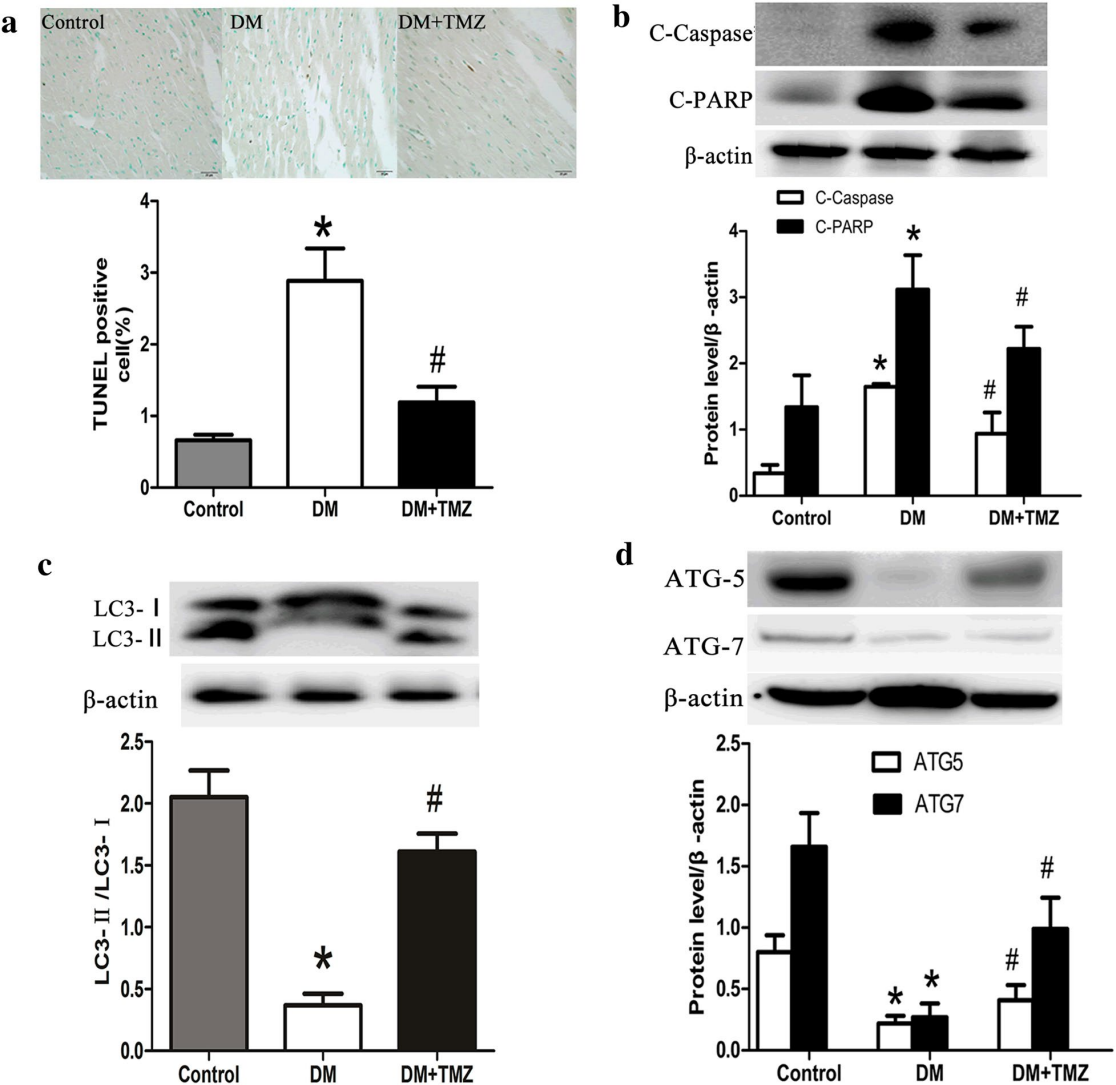
Autophagy and cardiac apoptosis in rats in the control, diabetes mellitus (DM), and diabetes + trimetazidine (DM + TMZ) groups. **a** Representative images and analysis of the TUNEL assay (*scale bar* 20 μm). **b** Representative western blot bands and analysis of C-Caspase 3/β-actin and C-PARP/β-actin. **c** Representative western blot bands and analysis of LC3-II/LC3-I. **d** Representative western blot bands and analysis of ATG5/β-actin and ATG7/β-actin. Data are mean ± SEM. ATG, autophagy [protein]. C-PARP, C–poly (ADP-ribose) polymerase. LC3, light chain 3 [protein]. TUNEL, terminal deoxynucleotidyl transferase dUTP nick end labeling. *p < 0.05 compared with control group; and ^#^ *p* < 0.05 compared with diabetes group

**Fig. 6:**
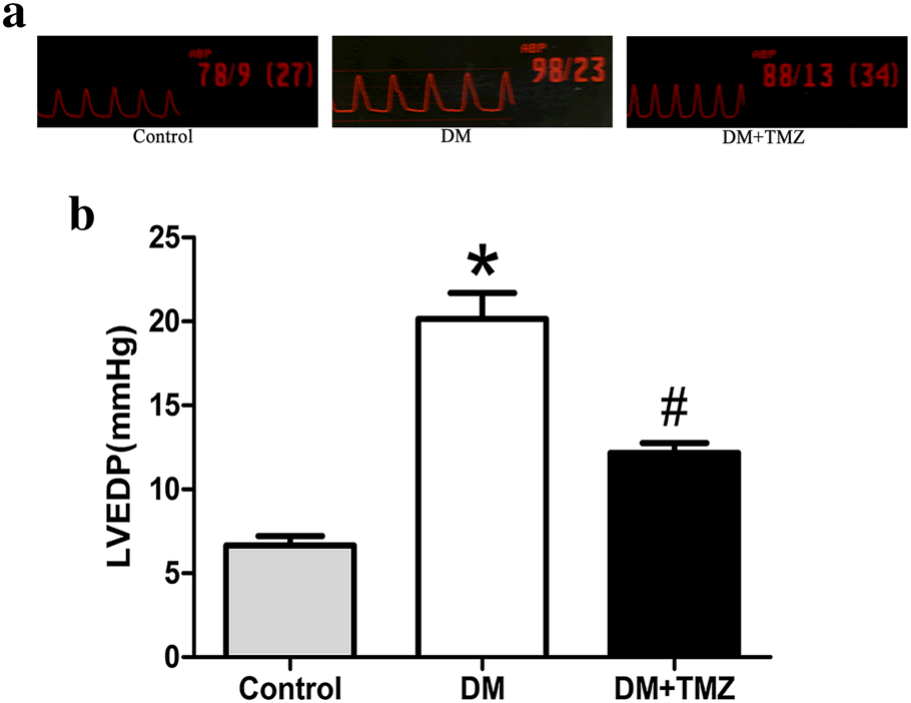
Left ventricular diastolic function in rats in the control, diabetes mellitus (DM), and diabetes mellitus + trimetazidine (DM + TMZ) groups at the end of the experiments. **a** shows pressure curves during cardiac catheterization, while **b** indicates left ventricular end diastolic pressure (LVEDP). Data are mean ± SEM; *n* = 5–6 per group. *p < 0.05 compared with control group; and ^#^ *p* < 0.05 compared with diabetes group

TUNEL staining demonstrated that TUNEL-positive cells were seldom identified in the control group, but numerous TUNEL-positive cells were observed in the diabetic group (Fig. 5a). Consistently, western analysis showed a significant increase in cleavage of Caspase-3 and Parp in the diabetic group (Fig. 6b).

#### Autophagy

The diabetic group revealed lower light chain 3 II (LC3-II) protein levels compared with the control (Fig. 5c), indicating a reduction in cardiac autophagy. Meanwhile, ATG5 and ATG7 (autophagy indicators) were significantly lower in the diabetic group than in the control group (Fig. 5d).

#### LV dysfunction assessed by catheterization

At the end of the experiment, LV end diastolic pressure was measured by cardiac catheterization. Diabetic rats showed higher LV pressure compared with the control (Fig. 6a). Thus, diastolic dysfunction developed and progressed during type 2 DCM.

#### PI3 K/AKT, MAPK and AMPK/Beclin1-Bcl2 pathways

**Fig. 7:**
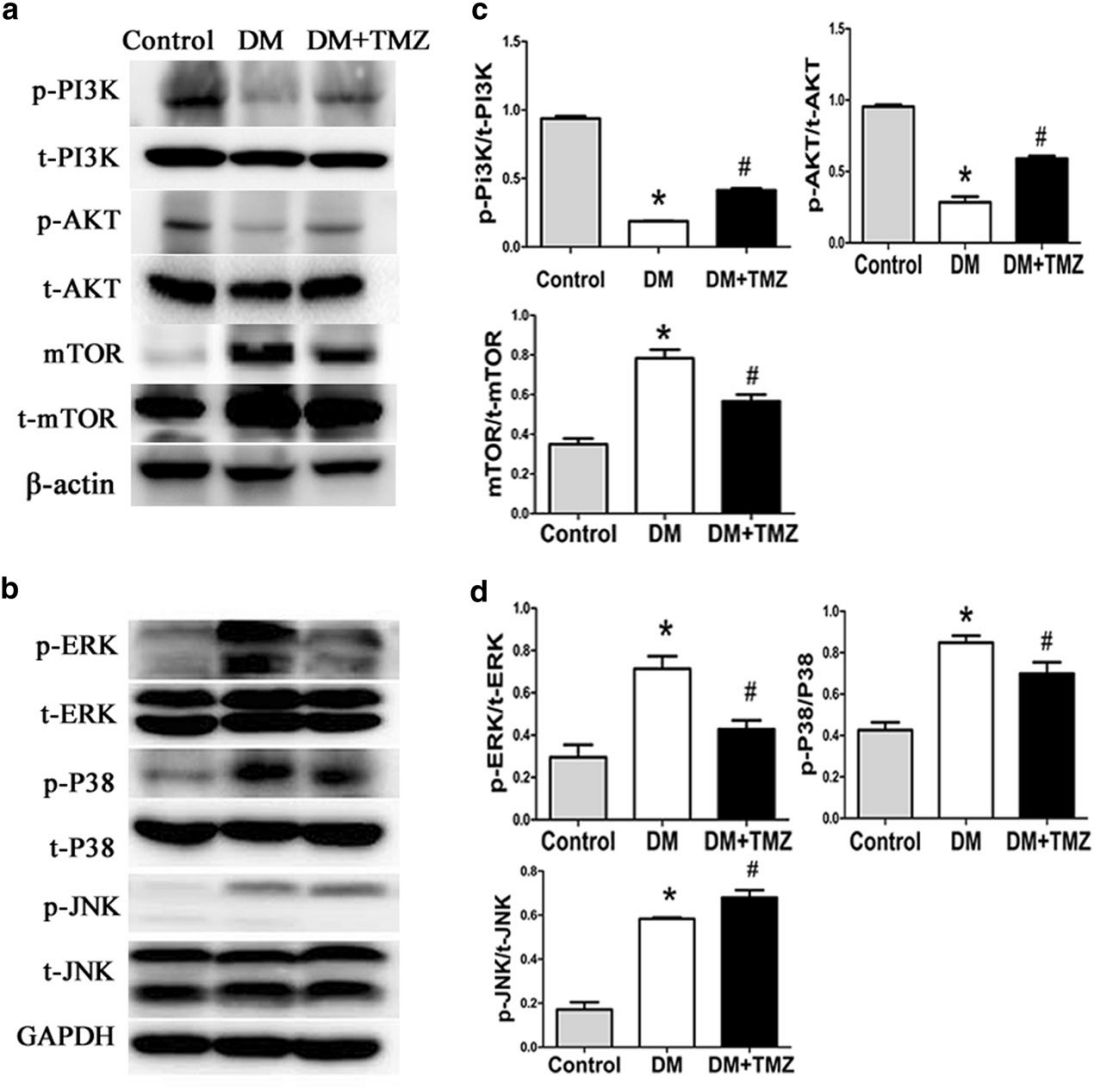
PI3 K/AKT and MAPK pathways in the heart of rats in the control, diabetes mellitus (DM), and diabetes mellitus + trimetazidine (DM + TMZ) groups. **a** Representative western blot bands of p-PI3 K, t-PI3K, p-AKT, t-AKT, mTOR and t-mTOR. **b** Representative Western blot bands of p-ERR, t-ERK, p-P38, t-P38, and p-JNK, t-JNK. **c** Representative western blot analysis of p-PI3 K/t-PI3K, p-AKT/t-AKT and mTOR/t-mTOR. **d** Representative western blot analysis of p-ERK/t-ERK, p-P38/t-P38, and p-JNK/t-JNK

**Fig. 8:**
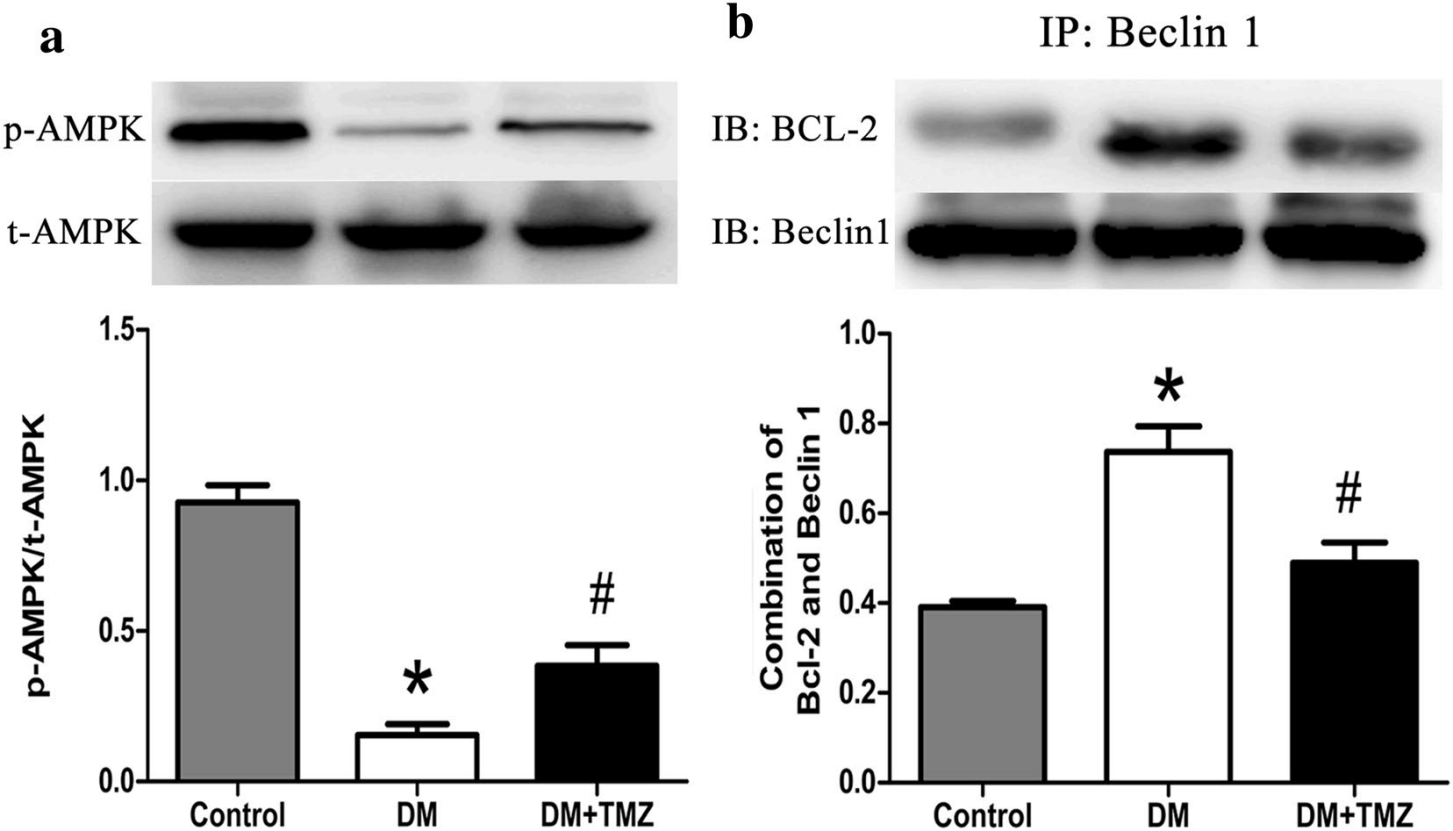
AMP-activated protein kinase (AMPK) phosphorylation and the interaction between the B cell lymphoma 2 (Bcl-2) and Beclin 1 proteins in the heart of rats in the control, diabetes mellitus (DM), and diabetes mellitus + trimetazidine (DM + TMZ) groups. **a** shows a western blot analysis of phosphorylated AMPK (p-AMPK)/total AMPK (t-AMPK), while **b** shows coimmunoprecipitation analysis of combination of the B-cell lymphoma 2 (Bcl-2) protein and the protein Beclin 1. Data are mean ± SEM; AMP, adenosine monophosphate. *p < 0.05 compared with control group; and ^#^ *p* < 0.05 compared with diabetes group

The levels of phosphorylated PI3 K, AKT and AMPK in diabetic group were significantly lower than in the control group (Figs. 7a, 8a). However, phosphorylated ERK, P38 and JNK were significantly elevated in the diabetic group. Meanwhile, enhanced interaction between Bcl-2 and Beclin1 was observed in the diabetic group (Figs. 7b, 8b).

### Trimetazidine reverses type 2 DCM

#### Trimetazidine ameliorated metabolism

At the end of the experiment, heart rate and heart weight were decreased in the diabetic group with trimetazidine treatment (Table 1). Total cholesterol, triglyceride, fasting blood glucose, and fasting insulin were significantly lower in the trimetazidine group than in the diabetic group (total cholesterol, 2.86 ± 0.53 vs. 5.72 ± 0.51 mmol/L; triglycerides, 2.90 ± 0.78 vs. 7.53 ± 0.92 mmol/L; fasting blood glucose, 15.33 ± 4.27 vs. 25.07 ± 1.21 mmol/L; fasting insulin, 3.37 ± 0.08 vs. 7.20 ± 0.51 mU/L, *p* < 0.05, respectively); the ISI and HDL-c were significantly increased in the trimetazidine group (ISI, −3.60 ± 0.85 vs. −5.19 ± 0.19, *p* < 0.05; HDL-c, 0.85 ± 0.07 vs. 0.33 ± 0.03 mmol/L, *p* < 0.05, respectively). These findings suggest that trimetazidine ameliorated metabolic disturbance and insulin resistance.

#### Trimetazidine improved glucose tolerance and insulin sensitivity

After the trimetazidine treatment, the IPGTT and IPITT revealed that levels of blood glucose in the trimetazidine group were significantly lower than those of the diabetic group (Fig. 2B1, B3). The trimetazidine group showed lower mean AUC on both IPGTT and IPITT than the diabetic group (Fig. 2B2, B4).

#### Trimetazidine reversed myocardial remodeling and reduced the deposition of collagen I and III content

After the trimetazidine treatment, the ratio of heart weight to body weight was reduced (2.88 ± 0.09 vs. 3.39 ± 0.07, *p* < 0.05, Fig. 3b). The trimetazidine group also showed lower CVF (4.75 ± 0.52 % vs. 11.04 ± 1.67 %, *p* < 0.05, Fig. 3d) and PVCA/LA ratio (8.37 ± 0.51 vs. 17.97 ± 2.66, *p* < 0.05, Fig. 3e) compared with the diabetic group. The deposition of collagen I and III content was significantly lower in the trimetazidine group than in the diabetic group after the trimetazidine treatment (Fig. 4).

#### Trimetazidine restored autophagy and protected against cardiac apoptosis

TUNEL staining demonstrated that TUNEL-positive cells were significantly lower in the trimetazidine group than in the diabetic group (Fig. 5a). A significant decrease in cleavage of Caspase-3 and Parp were observed in the trimetazidine group (Fig. 5b). After the trimetazidine treatment, LC3-II, ATG5, and ATG7 were increased (Fig. 5c, d). These data suggest that trimetazidine restored autophagy and protected against cardiac apoptosis.

#### Trimetazidine improved cardiac function

After the treatment, LV end diastolic pressure was significantly lower in the trimetazidine group than in the diabetic group (Fig. 6a), which confirmed the benefit effect of trimetazidine on improving LV diastolic dysfunction.

#### Trimetazidine regulated PI3 K/AKT, MAPK and AMPK/Beclin1-Bcl2 pathways

After the treatment, trimetazidine enhanced PI3 K, AKT, JNK and AMPK activity and disrupted the complex of Bcl-2 and Beclin1 in the trimetazidine group compared with diabetic group (Figs. 7, 8). However, trimetazidine inhibited ERK and P38 MAPK activity in the trimetazidine group compared with diabetic group (Fig. 7b).

## Discussion

In our present study, we found that early administration of trimetazidine could ameliorate diabetic cardiomyopathy. We showed this cardioprotective effect of trimetazidine was associated with decreased myocardial fibrosis, reduced cardiomyocyte apoptosis, and enhanced autophagic capacity. We demonstrated that trimetazidine alleviated myocardial fibrosis by modulating MAPK pathway. In addition, trimetazidine restored autophagy and protected against cardiac apoptosis mainly by activating AMPK and dissociating Beclin1 and Bcl-2. Thus, early administration of trimetazidine could be a good choice in the prevention of diabetic cardiomyopathy if applied at the very early stage of diabetes.

Diabetic cardiomyopathy, independent of coronary artery disease and hypertension, is defined as left ventricular dysfunction [14], and it is well established as one of the major causes of heart failure in diabetic patients. Since diabetic cardiomyopathy begins with a latent period clinically, treatment is often delayed and overlooked, resulting in poor prognosis. Thus, attention must be focused on the prevention of the disease. Therefore, we treated the rats with trimetazidine at the early stage of diabetes. We found that trimetazidine reversed myocardial remodeling and improved LV diastolic dysfunction. Therefore, we can conclude that early administration of trimetazidine could ameliorate diabetic cardiomyopathy.

Insulin resistance plays a causal role in physiopathology, appearance and nosogenesis of metabolic disorder in vivo [15]. It increases the risk of cardiovascular damage in diabetes patients [16] and is one of the important causes in the development of DCM [12]. After early administration of trimetazidine for 8 weeks, insulin resistance was ameliorated in our study. One of the mechanisms that trimetazidine ameliorated the insulin-resistant states possibly via restoring the blunted insulin signaling pathway-PI3 K/AKT. PI3 K/AKT, which is a key component in the signal transduction of insulin and plays a casual role in glucose and lipid metabolism [17], could promote GLUT4 translocation to the sarcolemma [18]. Therefore, we explored the changes in the PI3 K/AKT pathway. In our experiments, we found that the phosphorylation of PI3 K and AKT were decreased in type 2 diabetes rats model and trimetazidine restored PI3 K/AKT pathway. More recently, activation of AMPK has also been shown to trigger the translocation of GLUT4 [19]. Thus, AMPK was also explored in our study. We found AMPK was inhibited in an insulin-resistant state and unregulated after administration of trimetazidine in our study. Therefore, trimetazidine ameliorated the insulin-resistant states mainly by activating PI3 K/AKT and AMPK pathways.

A major player in the pathophysiology of DCM is myocardial fibrosis. Myocardial fibrosis is characterized by excessive deposition of collagen I and III [20]. In this study, diabetic rats showed prominent collagen accumulation in interstitial and perivascular areas on Masson trichrome and Picrosirius red staining. Treatment of trimetazidine reduced the aberrant interstitial and perivascular collagen accumulation, as well as total collagen content. Previous studies have shown that activated MAPK plays an essential role in cardiac fibrosis [12, 21]. Therefore, we explored the changes in the MAPK pathway. We found a generalized decrease in phosphorylation of ERK and P38 MAPK, suggesting the improvements in cardiac fibrosis were mainly attributed to the decreased activation of ERK and P38 MAPK.

In contrast to its adaptive nature in type 1 diabetes, in most HFD-induced type 2 diabetic models the inhibited cardiac autophagy appears to be maladaptive, and thus strategies to enhancing autophagy may be cardioprotective [22–24]. An increase in cardiac apoptotic cell death has also been demonstrated as a major event in the development of DCM [25, 26]. Therefore, autophagy and apoptosis were observed in our study. Inhibition of cardiac autophagy along with enhanced cardiomyocyte apoptosis were found in the insulin-resistant states. Trimetazidine therapy restored cardiac autophagic activities and protected against apoptosis. Inhibition of AMPK enhances the interaction between Bcl-2 and Beclin1 in type 1 diabetic mice, resulting in the alleviation of autophagy and the initiation of apoptosis [27]. Therefore, we explored the changes of AMPK and Bcl-2-Beclin1 complex in the insulin-resistance states. Our study found that not only AMPK activity was significantly inhibited but also the interaction between Bcl-2 and Beclin1 was enhanced in our type 2 diabetes rats model. However, trimetazidine restored AMPK activity and dissociated the interaction between Bcl-2 and Beclin1. Combined with previous studies, trimetazidine restored autophagy and protected against cardiac apoptosis mainly by activating AMPK and dissociating Beclin1 and Bcl-2.

## Conclusion

Early administration of trimetazidine could ameliorate diabetic cardiomyopathy by inhibiting myocardial fibrosis, reducing cardiomyocyte apoptosis, and enhancing autophagy. Therefore, trimetazidine may be a good choice in the prevention of diabetic cardiomyopathy if applied at the very early stage of diabetes.
