# Erratum to: Early administration of trimetazidine attenuates diabetic cardiomyopathy in rats by alleviating fibrosis, reducing apoptosis and enhancing autophagy

**DOI:** 10.1186/s12967-016-1068-5

**Published:** 2016-11-01

**Authors:** Lei Zhang, Wen-yuan Ding, Zhi-hao Wang, Meng-xiong Tang, Feng Wang, Ya Li, Ming Zhong, Yun Zhang, Wei Zhang

**Affiliations:** 1 Key Laboratory of Cardiovascular Remodeling and Function Research, Chinese Ministry of Education and Chinese Ministry of Public Health, Department of Cardiology, Qilu Hospital of Shandong University, Ji’nan, People’s Republic of China; 2 Department of Cardiology, Shandong Provincial Qianfoshan Hospital, Shandong University, Ji’nan, People’s Republic of China; 3 Department of Geriatric Medicine, Qilu Hospital of Shandong University, Ji’nan, People’s Republic of China; 4 Department of Emergency, Qilu Hospital of Shandong University, Ji’nan, People’s Republic of China

## Erratum to: J Transl Med (2016) 14:109 DOI 10.1186/s12967-016-0849-1

Unfortunately, the original version of this article [1] contained an error. The affiliations were incorrect. The correct author list and associated affiliations can be found in this erratum.
